# Nontuberculous Mycobacteria in Guadeloupe, Martinique, and French Guiana from 1994 to 2012

**DOI:** 10.1155/2013/472041

**Published:** 2013-12-18

**Authors:** Elisabeth Streit, Julie Millet, Nalin Rastogi

**Affiliations:** WHO Supranational TB Reference Laboratory, Tuberculosis & Mycobacteria Unit, Institut Pasteur de la Guadeloupe, Morne Jolivière, BP 484, 97183 Les Abymes, France

## Abstract

Nontuberculous mycobacteria (NTM) are ubiquitous environmental organisms able to cause severe opportunistic human infections. Their distribution patterns are subject to geographical variations. This study describes their isolation frequencies from clinical specimen in the three French overseas departments of the Americas, namely, Guadeloupe, Martinique, and French Guiana during 1994–2012. A total of 651 strains from as many patients (one isolate per species per patient) were analysed regarding regional isolation patterns and potential pattern changes over time. The *Mycobacterium avium* complex was the most common group of NTM in Guadeloupe and French Guiana. In Martinique it was the second most common after the rapidly growing mycobacteria. *M. fortuitum* was the most commonly isolated species in all three departments. Some species (*M. kansasii*, *M. xenopi*, and *M. terrae* complex) displayed a clear regional preference. Furthermore a change in isolation frequency was observed for *M. intracellulare* (increase) and *M. kansasii* (decrease) in Guadeloupe. In conclusion, marked regional differences in isolation frequencies of NTM species were observed in the study area. Results are discussed in context of variables such as study populations, risk factors, methodology employed, isolation from pulmonary versus sterile isolation sites (blood, urine, and CSF), and in vitro drug-susceptibility patterns.

## 1. Introduction

Aside from the obligate pathogens *Mycobacterium tuberculosis* and* M. leprae*, the genus *Mycobacterium* includes a high number of environmental organisms commonly referred to as nontuberculous (NTM) or atypical mycobacteria (the list of prokaryotic names with standing in nomenclature [1] lists 164 mycobacterial species and 13 subspecies). Environmental mycobacteria can be found in a wide range of aquatic and terrestrial habitats. They have been isolated from dusts, aerosols, and biofilms [2] and are frequently recovered from domestic or hospital water distribution systems [3]. A number of these environmental mycobacteria act as opportunistic pathogens causing a variety of diseases. Such infections are often linked to predisposing factors like immunodeficiency or underlying lung disease [4]. Apart from pulmonary infections, NTM can be implicated in skin disease, soft tissue infection, skeletal infections, lymphadenitis, foreign body-related infections (e.g., catheter-related infections), and bacteremia in AIDS patients amongst others [4, 5]. A considerable increase in NTM infections has been observed in the 1980s and early 1990s [6, 7]. While improved antiretroviral treatment has led to a decrease in the incidence of disseminated *Mycobacterium avium* complex infections in AIDS patients, the rate of pulmonary NTM infections is increasing [4]. In part, this increase is certainly attributable to better diagnostic tools and increased awareness to NTM disease. Nevertheless, NTM are important pathogens not least because their high level of natural antibiotic resistance renders them difficult to treat [4, 8]. It has been observed that there are marked regional differences regarding the rate at which a given NTM species is isolated from clinical samples [6, 9]. These geographic differences have recently been investigated for the first time at a global level by Hoefsloot et al. [9].

In this study we report the distribution of NTM in the three French overseas departments of the Americas, namely, Guadeloupe, Martinique, and French Guiana based on the results of 651 clinical NTM isolates obtained over the 19-year period from 1994 to 2012. The influence of liquid versus solid culture on the isolation frequency of NTM, as well as other variables such as study populations, risk factors, methodology employed, isolation from pulmonary versus sterile isolation sites, drug-susceptibility patterns, and so forth, was examined.

## 2. Methods

### 2.1. Patients and Specimens

The NTM strains were isolated from clinical specimen obtained from patients treated in Guadeloupe, Martinique, or French Guiana in the period of 1994–2012 and represent a complete collection of the clinical mycobacterial isolates of these three settings. In order to ensure the interpretability of the results, only one isolate per species per patient was retained for analysis. Isolates were cultured exclusively on solid media (LJ, 7H10, and 7H11 medium), except in Guadeloupe where BACTEC MGIT960 liquid culture system (Becton-Dickinson, Franklin Lakes, NJ, USA) was introduced in January 2007. From 1994 to 1995, NTM isolates were identified by growth and cultural characteristics and routine biochemical tests. Starting in 1996 the AccuProbe assay (Gen-Probe Inc., San Diego, CA, USA) was used, allowing for the differentiation between *M. avium* and *M. intracellulare* and facilitating the identification of *M. kansasii* and *M. gordonae*, although biochemical tests remained in use for the identification of other species. Furthermore, PCR-restriction fragment length polymorphism analysis (PRA) of *hsp65* gene [11] was used as a complementary identification technique for research purposes in parallel. In late 2006, all the three methods were replaced by the GenoType Mycobacteria CM/AS assays (Hain Lifescience GmbH, Nehren, Germany).

### 2.2. Drug-Susceptibility Testing

Drug-susceptibility testing was performed as described previously [10]. Briefly, the isolates were tested for susceptibility to drugs on 7H11 agar medium at the following concentrations: isoniazid (INH, 0.2 and 1 *μ*g/mL), rifabutin (RBT, 1 *μ*g/mL), rifampicin (RIF, 1 and 15 *μ*g/mL), ethambutol (EMB, 7.5 *μ*g/mL), D-cycloserine (D-CS, 30 *μ*g/mL), ethionamide (ETH, 10 *μ*g/mL), clofazimine (CLOFA, 1 *μ*g/mL), streptomycin (SM, 2 *μ*g/mL), amikacin (AMIK, 4 and 20 *μ*g/mL), kanamycin (KANA, 6 *μ*g/mL), ofloxacin (OFLO, 1.5 and 5 *μ*g/mL), ciprofloxacin (CIPRO, 1.5 and 5 *μ*g/mL), sparfloxacin (SPARFLO, 1.5 *μ*g/mL), and clarithromycin (CLARIT, 32 *μ*g/mL).

### 2.3. Statistical Analysis. 

STATA version 12.1 was used for statistical analysis. Percentages were compared using the chi-square test;  *P* values of ≤0.05 were considered statistically significant. Trend analysis was done using the nptrend command of STATA which performs the nonparametric test for trend across ordered groups developed by Cuzick [12]. To facilitate the identification of long-term changes, data were furthermore divided into three groups of five years (1994–1998, 1999–2003, and 2004–2008) and one group of four years (2009–2012) and analysed using the average of the variable of interest for each group. This approach permitted to even out yearly variations in isolation frequencies. Note that a specific classification was used to assess the impact of the implementation of the MGIT culture system in 2007 by splitting the data in groups: 1994–2006 (pre-MGIT) versus 2007–2012 (MGIT).

## 3. Results 

A total of 651 isolates of atypical mycobacteria were obtained from patients in Guadeloupe, Martinique, and French Guiana (results are summarised in Tables 1–3). Overall, 595/651 (91.3%) of the isolates were identified to species level. Based on the information recorded in the laboratory register about number of days to positive mycobacterial growth, the remaining 56 unidentified isolates were tentatively classified as rapidly growing (RGM; *n* = 14; 2.15%) and slowly growing (*n* = 21; 3.22%) species or marked as mycobacteria other than *M. tuberculosis* (MOTT; *n* = 21; 3.22%) when this information was not available.

During the 19 years of data collection, a significant trend towards higher mean age of patients was observed (particularly in Martinique; *P* = 0.003), the mean age of the patients being 53 years (range of 48 years in French Guiana to 58 years in Martinique). Gender and HIV serology information was available for 615 and 225 patients, respectively, with a male to female sex ratio of 1.63 and a positive HIV serology in 129/225 (57.3%) cases.

The majority of the clinical specimens (75%) were pulmonary samples. The proportion of patients from which only extrapulmonary samples had been obtained differed significantly between the three departments, ranging from 16% in French Guiana to 30% in Martinique (Guadeloupe: 20%; *P* = 0.003). Clinical specimen obtained by expectoration were the most common (*n* = 204), followed by gastric intubations (*n* = 188) and blood samples (*n* = 86). Note that the great majority of the blood samples yielded isolates identified as *M. avium* (*n* = 40/86) or MAC (*n* = 35/86). Other species isolated from blood samples were *M. intracellulare* (*n* = 4), *M. simiae* (*n* = 6), and *M. kansasii* (*n* = 1).

**Figure 1:**
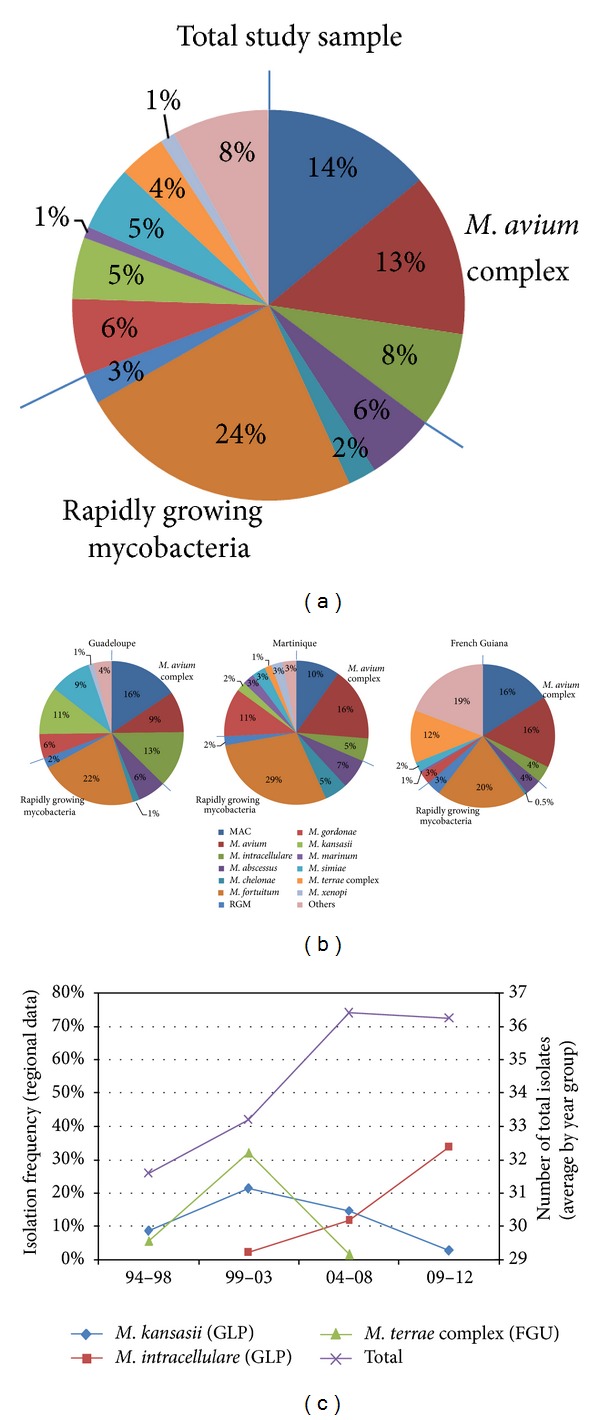
Distribution patterns of nontuberculous mycobacteria isolated from clinical samples in the three French departments of the Americas from 1994 to 2012. (a) Total study sample *n* = 651 strains (one isolate per species per patient); (b) distribution of strains per department, that is, Guadeloupe *n* = 270, Martinique *n* = 194, and French Guiana *n* = 187; (c) mean isolation frequency for *M. kansasii* and *M. intracellulare* in Guadeloupe and for *M. terrae* complex in French Guiana versus total isolates during study periods of 1994–1998, 1999–2003, 2004–2008, and 2009–2012 (numbers are shown as average number per year on secondary *x*-axis; mean values are illustrated to counterbalance the fact that the last interval consisted of four years).

Regarding the distribution of the various NTM species, twenty species were observed (Tables 1–3, Figure 1(a)). Species belonging to the *Mycobacterium avium* complex (MAC) accounted for 230/651 or 35.3% of all the isolates, making them the most frequently isolated group of atypical mycobacteria in this study. They were equally prevalent in Guadeloupe, Martinique, and French Guiana, constituting 37%, 31%, and 36% of the strains, respectively (Table 1; Figure 1(b)). Of the 230 MAC isolates, 138 (60%) were identified to species level; in general, *M. avium* was more frequently encountered than *M. intracellulare* (63% versus 37% among those identified at species level; *M. avium n* = 87, *M. intracellulare n* = 51). However, the proportion of *M. avium* was higher in Martinique and French Guiana (76 and 81%, resp.) than in Guadeloupe (42%). In contrast, *M. intracellulare* was isolated more frequently as a percentage of the total isolates in Guadeloupe (13% versus 5.1% in Martinique and 3.7% in French Guiana; *P* = 0.001) than *M. avium* (9.3% in Guadeloupe versus 16% and 16%, resp., in Martinique and French Guiana; *P* = 0.037). Note that *M. intracellulare* was increasingly isolated in Guadeloupe during the second half of this study, 12% of all strains in 2004–2008 versus 34% in 2009–2012 (*P* = 0.002; Table 1, Figures 1(b) and 1(c)).

Rapidly growing mycobacteria (RGM) made up 34% of the isolates and a significant difference was observed regarding the percentage of RGM isolates in the three departments (Figure 1(b); French Guiana 30%, Guadeloupe 33%, and Martinique 45%; *P* = 0.004). Among these, *M. fortuitum* was the most commonly isolated species both in the global sample (153/651, 23.5%), and in the three departments (Martinique 29%, Guadeloupe 22%, and French Guiana 20%). It was followed by *M. abscessus* (*n* = 37) and *M. chelonae* (*n* = 15) which were the most commonly isolated RGM.

Significant differences were observed for *M. gordonae*, *M. chelonae* (RGM), and *M. simiae* in the three departments (Tables 1–3, Figure 1(b)). The first two were more prevalent in Martinique (11% and 5.2%, resp.; *P* = 0.004 and 0.005) than in Guadeloupe (5.6%; 1.5%) or French Guiana (2.7%; 0.5%) while *M. simiae* was more widespread in Guadeloupe (9.3%) than in the other two departments (3.1% and 2.1% for Martinique and French Guiana; *P* = 0.001). Interestingly, *M. kansasii *was only isolated in the two island settings with the great majority of the isolates (29/33) coming from Guadeloupe, but not in French Guiana (Table 2). Nonetheless, its isolation frequency somewhat diminished after 2008, stabilising at one isolate per year with the exception of 2010 when no *M. kansasii* strains were isolated in any of the departments. On the same line, *M. xenopi *was only isolated in patients from Guadeloupe (3/8) and Martinique (5/8) but but not in patients from French Guiana. In contrast, *M. terrae* complex strains were primarily isolated from patients in French Guiana (22/25) and none in Guadeloupe; interestingly a great majority (*n* = 20) was obtained in 3-year period from 2000 to 2002 with a peak of 10 isolates in 2001 (all from French Guiana; Figure 1(c)).

We also investigated if the introduction of liquid culture system BACTEC MGIT 960 in January 2007 in Guadeloupe influenced the proportion of positive cultures, based on available data on pathological samples received between 1994 and 2006 (*n* = 31307) versus samples received from 2007 to 2012 (*n* = 10499). It was observed that the introduction of the MGIT system significantly increased the percentage of mycobacteria positive specimen (1648/31307, 5.3%, in 1994–2006 to 778/10499, 7.4%, in 2007–2012; *P* < 0.0001). However, the liquid culture by MGIT system essentially benefited the isolation of NTM. Indeed the ratio of total diagnosed cases of *M. tuberculosis* per total number of specimen was unaffected by the new culture method (407/31307, 1.3%, in 1994–2006 versus 133/10499, 1.3%, in 2007–2012; *P* = 0.7941) as opposed to a significant increase in NTM isolation (201/31307, 0.6%, in 1994–2006 versus 122/10499, 1.2%, in 2007–2012; *P* < 0.0001). Lastly, the introduction of MGIT culture in Guadeloupe significantly reduced the proportion of microscopy+/culture− cases among all positive samples (556/1648, 33.7%, in 1994–2006 versus 122/778, 15.7%, in 2007–2012; *P* < 0.0001) to the benefit of microscopy−/culture+ cases (398/1648, 24.2%, in 1994–2006 versus 301/778, 38.7%, in 2007–2012; *P* < 0.0001), clearly showing the benefit of liquid over solid culture for mycobacterial isolation, in addition to shorter turn-around time to culture positivity.

Last but not least, the drug-susceptibility patterns (defined as ≤10% of all isolates displaying in vitro resistance) essentially confirmed previous observations on highly variable natural drug resistance of NTM (Tables 1–3; [10]). *M. abscessus* was the most drug-resistant organism whereas *M. xenopi* and *M. kansasii* were the most drug-susceptible organisms, while other isolates showed varying drug-susceptibility patterns. Among drug families, the largest spectrum of in vitro activity was shown by macrolide drug clarithromycin, fluoroquinolones, D-cycloserine, and clofazimine; intermediate activity was shown by rifamycins, ethambutol, ethionamide, and aminoglycosides, while isoniazid was the least active. Seeing their pharmacokinetic properties and lesser side effects, both clarithromycin (with activity against MAC including *M. avium* and *M. intracellulare*, *M. chelonae*, *M. gordonae, M. kansasii, M. simiae, M. terrae* complex, *M. xenopi, M. flavescens, M. interjectum, M. malmoense, *and *M. scrofulaceum*) and quinolones (with activity against unidentified RGMs,* M. fortuitum*, *M. gordonae*, *M. xenopi, M. flavescens, *and* M. malmoense*) are among potentially active drugs to treat NTM infections.

## 4. Discussion

To the best of our knowledge, this is the first report of the distribution of nontuberculous mycobacteria in the French overseas departments of the Americas. Other studies have discussed the NTM species distribution on a multicountry [7] or global level [9]. In most regions of the world, the *Mycobacterium avium* complex which causes disseminated infection in AIDS patients [4] is the most frequently observed NTM among patient isolates [6, 7, 9]. It was found to be highly prevalent in all three departments and accounted for 31–37% of the isolates, which corresponds to the rates reported for Europe and South America by Hoefsloot et al. [9]. Given that almost half of the *M. avium* isolates (*n* = 40/87) and about a third of the MAC isolates (*n* = 35/92) were obtained from blood samples, it is evident that these NTM are frequently implicated in actual disease in contrast to isolates from pulmonary samples that may indicate transient colonisation following contact with environmental NTM strains.

Even though HIV serology was not reported systematically for a majority of the patients, the association of HIV and MAC/*M. avium* coinfections was confirmed by our data (Table 1; 43/4 and 40/8 isolates, resp., from HIV+/HIV− patients). However, no such association was observed for *M. intracellulare* (5/5 isolates from HIV+/HIV− patients, resp.). Our previous results indicated that there was no privileged ecological niche for *M. avium* infecting AIDS patients in the study area [13], a finding that would merit further investigations. On the contrary, no significant association of isolation frequency and HIV status was observed for *M. simiae* in the present study (*P* = 0.2035) despite the fact that *M. simiae* has been linked to infections of terminally ill AIDS patients in Guadeloupe in the past [14, 15]. Lastly, *M. intracellulare* was isolated more frequently in Guadeloupe than *M. avium*, which is rather uncommon globally [9]. Moreover, a distinct increase in the isolation frequency of *M. intracellulare* in Guadeloupe was observed during the second half of the study period. Considering the higher pathogenicity of *M. intracellulare* compared to *M. avium* in HIV negative persons [16], it would be important to investigate whether the comparative abundance of* M. intracellulare* in Guadeloupe is due to the existence of an ecological niche or infection source specific for this island or if it is a mere artefact of the local health care practices.

The rapid growers were more frequently isolated from HIV− patients; for example, none of the 10 *M. abscessus* isolates obtained from patients with known HIV serology in our study implicated an HIV+ patient. For *M. chelonae* and *M. fortuitum* the proportion was 1/5 (*P* = 0.0412) and 15/32 (*P* = 0.001), respectively. *M. gordonae* too was more commonly isolated from HIV− patients (*P* = 0.0050). Contrary to Guadeloupe and French Guiana, rapid growers were more frequently isolated than MAC strains in Martinique (45% of all isolates), a proportion close to the global maximum values of 50% in Taiwan [9]. (Of note, a high prevalence of RGM is one of the key characteristics encountered in Asian NTM epidemiology [17]). Even though the percentage of RGM in the other two departments is significantly lower (around 30%) than that observed in Martinique, these values are still rather high compared to the global average of 10–20% RGM isolates reported by Hoefsloot et al. [9]. In all three departments *M. fortuitum* was the most commonly isolated species of RGM. As this species is usually susceptible to several oral antibiotics [8] and not prone to rapid disease progression [18], the prognosis for patients is generally good. Also, the clinical significance of *M. fortuitum* isolates needs to be evaluated carefully, especially if they have been obtained from respiratory specimen [18]. A previous study from 1993 to 1999 found no evidence for nosocomial *M. fortuitum* outbreaks in the French departments of the Americas and questioned the clinical significance of its isolation from respiratory specimens [19]. Another study conducted in a Korean hospital found that of 26 patients with two or more respiratory specimens positive for this species (156 patients had only one positive result) only one required specific antibiotic therapy for *M. fortuitum* pulmonary infection [20].

As observed on a global scale [9], marked geographic differences regarding the prevalence of other NTM species were observed; for example, *M. terrae* complex was almost exclusively observed in French Guiana while *M. xenopi* and *M. kansasii* were solely observed in Guadeloupe and Martinique. The absence of *M. xenopi* in French Guiana is in agreement with previously reported data from South America [7, 9]. The lack of *M. kansasii* isolates in French Guiana, however, disagrees with earlier findings reporting an elevated prevalence of 20% in South America [9] and 12% in Brazil [7] for this species.

In continental France, *M. xenopi* was the third most common species after MAC and *M. gordonae* accounting for 8% of the isolates in 2008 [9]. It has been isolated particularly frequently in the English Channel region [9]. Another study on NTM isolates from 1991 to 1996 in several countries reported *M. xenopi* to be the second most common in France after MAC isolates (21.3% of the total isolates [7]). Hoefsloot et al. [9] noted that although *M. xenopi* was the third most frequent species in their survey, its isolation was limited to distinct geographical regions, namely, Europe and eastern Canada, suggesting the existence of a specific ecological niche for this species. Thus the prevalence of *M. xenopi* in Guadeloupe and Martinique (1.1% and 2.6%, resp.) is much lower than the rates observed in Europe (14%) and North America (12%) in 2008 [9] or in France from 1991 to 1996 [7].

*M. kansasii* accounted for 5% [9] to 11.4% [7] of NTM isolates in metropolitan France, being particularly frequent in and around Paris [9]. Its prevalence in Guadeloupe (11%) and to lesser extent in Martinique (2.1%) is in line with these observations. However, significant changes in the isolation frequency of *M. kansasii* were observed in Guadeloupe during the study period (*P* = 0.008); by year groups its proportion surged from 8.6% in 1994–1998 to 21% in 1999–2003 and has steadily declined since then. *M. kansasii* still accounted for 15% of the isolates from Guadeloupe in 2004–2008, before plummeting to 2.7% in 2009–2012 (Figure 1(c)). Although it was rather uncommon in the pre-AIDS era [21], *M. kansasii* became the second most common cause of pulmonary NTM disease in HIV+ patients in USA in early nineties [8]. Whether the declining isolation frequency of this species might reflect the advances made in terms of efficient antiretroviral therapy of all HIV+ patients in the French departments remains a debatable question.

As the AccuProbe assay did not distinguish *M. abscessus *from* M. chelonae*, the distinction between these 2 species did not appear in our official identification results until 2007. However, complementary PRA of the *hsp65* gene revealed that most of the isolates (*n* = 22/28) identified as *M. chelonae* complex between 1994 and 2007 were indeed *M. abscessus*. Given that *M. abscessus* is one of the most drug-resistant and difficult-to-treat organisms, this is an important distinction to make [8, 22, 23]. Recent findings revealed, for example, that *M. abscessus* but not *M. chelonae* disposes of an *erm* gene that confers inducible macrolide resistance [24]. *M. abscessus* is most renowned for causing skin and soft tissue infections following trauma or surgery [23]. It has also been implicated in pulmonary disease [8] especially in cystic fibrosis patients [23]. Unfortunately, there are no drug regimens of proven or predictable efficacy for treating *M. abscessus* lung disease [8].

Lastly, fifty-six (8.6%) of the isolates could not be identified at the species level. Hence a more precise characterisation of these isolates by 16S rDNA and *hsp65* sequencing (proposed as suitable by Ferdinand et al. [25]) seems worthwhile.

In summary, this study describes the distribution pattern of NTM species isolated from patients in Guadeloupe, Martinique, and French Guiana illustrating marked regional differences. An important limitation of this work is the scarcity of supporting information on the patients (like HIV status, underlying medical conditions, etc.) which precludes an in-depth analysis of potential risk factors for NTM infection in the studied area. Nevertheless we were able to reconfirm the association of *M. avium* infections and HIV while showing that RGM are of minor importance in this patient group. We also observed an intriguing abundance of *M. intracellulare* in Guadeloupe which merits further investigation. Lastly, except for *M. intracellulare* and *M. kansasii*, no specific trends of the isolation frequency were observed for a given species. Instead short-lived prevalence peaks were observed for some species, the most notable example being *M. terrae* complex, peaking between 2000 and 2002 in French Guiana. Of note, this species has not been isolated from patients in any of the three departments after 2004. According to the American Thoracic Society, *M. terrae* complex is of low pathogenicity and most often isolated as a contaminant [8]. It is therefore possible that the sudden surge of *M. terrae* complex isolates in 2001 is at least partly due to contamination of the samples. As has been stated for *M. fortuitum*, the clinical relevance of NTM isolates needs careful evaluation. Caution must be taken so as not to confound colonisation by NTM with NTM infection, in particular if the isolates are cultured just once from a single pulmonary specimen.

## Figures and Tables

**Table 1:** Isolation patterns of the two most frequently isolated NTM species, the *M. avium* complex and rapidly growing mycobacteria.

Parameters	Total	*M. avium* complex				Rapidly growing mycobacteria				
		MAC	*M. avium *	*M. intracellulare *	Total MAC	*M. abscessus *	*M. chelonae *	*M. fortuitum *	RGM	Total RGM^b, c, d^
Years										
1994–1998	158	39	23	1	**62**	7	1	35	5	**49** ^ b^
1999–2003	166	15	25	3	**43**	12	1	41	4	**60** ^b,c^
2004–2008	182	34	23	15	**72**	10	7	38	2	**58** ^ d^
2009–2012	145	4	16	32	**52**	8	6	39	3	**56**
	**651**	**92**	**87**	**51**	**230**	**37**	**15**	**153**	**14**	**223**
Age										
≤19	18	2	0	1	**3**	3	1	3	1	**8**
20–39	122	27	38	6	**71**	6	0	16	0	**22**
40–54	166	24	26	15	**65**	7	3	35	4	**50** ^ b^
55–69	125	5	11	17	**33**	8	3	38	2	**52** ^ c^
≥70	130	8	5	10	**23**	10	7	41	6	**65** ^ d^
N/A	90	26	7	2	**35**	3	1	20	1	**26** ^ b^
Sex ratio	1.63	1.71	1.90	1.43	**1.71**	0.76	0.67	1.38	13.0	**1.29**
(M/F)	(380/233)	(48/28)	(55/29)	(30/21)	(133/78)	(16/21)	(6/9)	(83/60)	(13/1)	(120/93)
NA	—	16	3	—	**19**	—	—	10	—	**10**
HIV (pos/neg)^a^	129/96	43/4	40/8	5/5	88/17	0/10	1/5	15/32	1/2	17/49
Department										
GLP	270	43	25	34	**102**	17	4	59	6	**87** ^ d^
MTQ	194	19	32	10	**61**	13	10	56	3	**84** ^b,c^
GUF	187	30	30	7	**67**	7	1	38	5	**52** ^ b^
Drug-susceptibility pattern^e^ (nb tested)	—	D-CS, CLOFA, RBT, CLARIT (*n*, 74)	D-CS, CLOFA, CLARIT (*n*, 80)	RIF 15, D-CS, CLOFA, CLARIT (*n*, 46)	—	None	CLARIT (*n*, 15)	AMIK, OFLO 1.5, CIPRO 1.5, SPARFLO (*n*, 145)	OFLO 1.5, CIPRO 1.5, SPARFLO (*n*, 13)	—

^a^For patients with known HIV serology; ^b^ *M. flavescens *isolate comprised; ^c^ *M. aurum* isolate comprised; ^d^ *M. mucogenicum* isolate comprised.

NA: not available; GLP: Guadeloupe; MTQ: Martinique; GUF: French Guiana.

^e^Drug-susceptibility pattern for a given NTM species was defined as ≤10% of all isolates displaying in vitro resistance [10]; AMIK: amikacin; CIPRO: ciprofloxacin; CLARIT: clarithromycin; CLOFA: clofazimine; D-CS: D-cycloserine; OFLO: ofloxacin; RBT: rifabutin; RIF: rifampin; SPARFLO: sparfloxacin. Note that drug concentration is mentioned for drugs screened at 2 different concentrations.

**Table 2:** Isolation patterns of regularly encountered NTM.

Parameters	Total	*M. gordonae *	*M. kansasii *	*M. marinum *	*M. simiae *	*M. terrae *complex	*M. xenopi *
Years							
1994–1998	158	9	9	2	11	2	3
1999–2003	166	14	11	2	5	22	2
2004–2008	182	8	10	1	10	1	2
2009–2012	145	10	3	1	9	0	1
	**651**	**41**	**33**	**6**	**35**	**25**	**8**
Age							
≤19	18	1	0	1	0	2	0
20–39	122	4	8	1	2	5	0
40–54	166	8	12	1	7	6	4
55–69	125	14	4	1	6	5	1
≥70	130	12	6	1	6	5	1
NA	90	2	3	1	14	2	2
Sex ratio	1.63	1.22	1.21	2	1.2	3.6	—
(M/F)	(380/233)	(22/18)	(17/14)	(4/2)	(18/15)	(18/5)	(8/0)
NA	—	1	2	—	2	2	—
HIV (pos/neg)^a^	129/96	3/11	5/2	NA	9/3	2/2	0/1
Department							
GLP	270	15	29	0	25	0	3
MTQ	194	21	4	5	6	3	5
GUF	187	5	0	1	4	22	0
Drug-susceptibility pattern^b^ (nb tested)	—	OFLO 5, EMB, D-CS, CIPRO 1.5, SPARFLO, CLARIT (*n*, 38)	ETH, RIF 1, EMB, CLOFA, RBT, CLARIT (*n*, 30)	ETH, RIF 15, EMB, DCS (*n*, 6)	CLOFA, CLARIT (*n*, 32)	ETH, RIF 15, EMB, CLOFA, RBT, CLARIT (*n*, 25)	ETH, INH 1, OFLO, EMB, D-CS, KANA, AMIK 4, CLOFA, RBT, CIPRO 1.5, SPARFLO, CLARIT (*n*, 6)

^a^For patients with known HIV serology.

NA: not available; GLP: Guadeloupe; MTQ: Martinique; GUF: French Guiana.

^b^Drug-susceptibility pattern for a given NTM species was defined as ≤10% of all isolates displaying in vitro resistance [10]; AMIK: amikacin; CIPRO: ciprofloxacin; CLARIT: clarithromycin; CLOFA: clofazimine; D-CS: D-cycloserine; OFLO: ofloxacin; RBT: rifabutin; RIF: rifampin; SPARFLO: sparfloxacin. Note that drug concentration is mentioned for drugs screened at 2 different concentrations.

**Table 3:** Isolation patterns of rarely isolated NTM species.

Parameters	*M. aurum* ^ a^	*M. flavescens* ^ a^	*M. genavense/ M. triplex* ^ b^	*M. interjectum *	*M. malmoense *	*M. muco-genicum *	*M. scrofulaceum *	*M. species* (slow growing)
Total	1	2	1	3	1	1	4	42
Isolation year(s)	2000	1995, 2002	2009	2007 (2), 2012	2009	2008	2008 (3), 2012	All (except 1999, 2004, 2009)
Mean age	63	NA, 41	34	73	24	81	55	52
Sex (M/F)	0/1	2/0	1/0	3/0	1/0	0/1	3/1	30/7
Origin	MTQ	MTQ, GUF	GLP	MTQ (1), GUF (2)	GUF	GLP	MTQ (1), GUF (3)	GLP (9), MTQ (3), GUF (30)
Drug susceptible to^c^	RIF 15, EMB, D-CS, CLOFA	OFLO 1,5 (2/2), EMB (2/2), AMIK 4 (2/2), RBT (2/2), SPARFLO (1/1), CLARIT (1/1)	No data	ETH (3/3), RIF 1 (3/3), D-CS (3/3), CLOFA (3/3), RBT (3/3), CLARIT (3/3), AMIK 20 (2/3)	ETH, RIF 1, SM, OFLO 1,5, EMB, D-CS, KANA, AMIK 4, CLOFA, RBT, CIPRO 1,5, SPARFLO 1,5, CLARIT	No data	ETH (4/4), RIF 15 (4/4), D-CS (2/3), AMIK 20 (2/4), CLOFA (4/4), CLARIT (4/4)	—

^a^RGM, included in total RGM listed in Table 1.

^b^Distinction between these 2 closely related species was not made.

NA: not available; GLP: Guadeloupe; MTQ: Martinique; GUF: French Guiana.

^c^Drug-susceptibility patterns were not generalized as mentioned for other species in Tables 1 and 2 (defined as ≤10% of all isolates displaying in vitro resistance [10]); instead, the results are given per strain (numbers in brackets for more than 2 strains). AMIK: amikacin; CIPRO: ciprofloxacin; CLARIT: clarithromycin; CLOFA: clofazimine; D-CS: D-cycloserine; OFLO: ofloxacin; RBT: rifabutin; RIF: rifampin; SPARFLO: sparfloxacin. Note that drug concentration is mentioned for drugs screened at 2 different concentrations.
